# Two-dimensional magnetic monopole gas in an oxide heterostructure

**DOI:** 10.1038/s41467-020-15213-z

**Published:** 2020-03-12

**Authors:** L. Miao, Y. Lee, A. B. Mei, M. J. Lawler, K. M. Shen

**Affiliations:** 1 Laboratory of Atomic and Solid State Physics, Department of Physics, Cornell University, Ithaca, NY 14853 USA; 2 School of Applied and Engineering Physics, Cornell University, Ithaca, NY 14853 USA; 3 Department of Materials Science and Engineering, Cornell University, Ithaca, NY 14853 USA; 4 Department of Physics, Binghamton University, Vestal, NY 13850 USA; 5 Kavli Institute at Cornell for Nanoscale Science, Ithaca, NY 14853 USA

**Keywords:** Magnetic properties and materials, Surfaces, interfaces and thin films

## Abstract

Magnetic monopoles have been proposed as emergent quasiparticles in pyrochlore spin ice compounds. However, unlike semiconductors and two-dimensional electron gases where the charge degree of freedom can be actively controlled by chemical doping, interface modulation, and electrostatic gating, there is as of yet no analogue of these effects for emergent magnetic monopoles. To date, all experimental investigations have been limited to large ensembles comprised of equal numbers of monopoles and antimonopoles in bulk crystals. To address these issues, we propose the formation of a two-dimensional magnetic monopole gas (2DMG) with a net magnetic charge, confined at the interface between a spin ice and an isostructural antiferromagnetic pyrochlore iridate and whose monopole density can be controlled by an external field. Our proposal is based on Monte Carlo simulations of the thermodynamic and transport properties. This proposed 2DMG should enable experiments and devices which can be performed on magnetic monopoles, akin to two-dimensional electron gases in semiconductor heterostructures.

## Abstract (web summary)

Heterostructure interfaces have physical properties distinct from bulk materials, providing the basis for many electronic devices. Miao et al. propose a spin ice heterostructure that can host a two-dimensional gas of emergent magnetic monopoles with a net magnetic charge.

## Introduction

Similar to electrons in a metal, monopoles in spin ice compounds^1,2^ are believed to be unconfined, interact via a magnetic analogue of Coulomb’s law^3^, and form magnetic currents in magnetic fields^4^. While exotic phases of matter such as fractional quantum Hall states^5^ or Wigner crystals^6^ can be realized in electronic systems, a natural question is whether magnetic monopoles can also exhibit rich behaviors if their charge and dimensionality can be controlled in a likewise fashion to those of electrons. However, to the best of our knowledge, all the current experiments have been restricted to bulk spin ice systems with equally populated monopoles and antimonopoles^4,7–13^. Here we propose such a platform, a heterointerface between a spin ice (R_2_Ti_2_O_7_) (R = Ho, Dy) and an antiferromagnet (R_2_Ir_2_O_7_) in the pyrochlore family. At the boundary, the exchange field between the iridate and the spin ice has a non-zero flux flowing toward/away from the spin ice, resulting in a charged 2DMG in the adjacent layers of the spin ice. Such boundary conditions are reminiscent of the polar discontinuity at the interface between LaAlO_3_ and SrTiO_3_ which hosts a two-dimensional electron gas^14,15^, and inspired by recent theoretical proposals suggesting the surface crystallization of magnetic monopole and antimonopoles at the material/vacuum interface of spin ice thin films^16,17^. Such a heterostructure represents an example of a magnetically charged monopole system, providing a unique opportunity to investigate their properties. In the following, we begin by describing the proposed heterostructure where such a 2DMG can be formed. We then investigate its transport and thermodynamics properties, and demonstrate that its properties can be modified by an external magnetic field. Finally, we demonstrate that the 2DMG remains robust even when including longer-ranged dipolar interactions.

## Results

### Spin structures of R_2_Ti_2_O_7_/R_2_Ir_2_O_7_ heterostructures

**Fig. 1:**
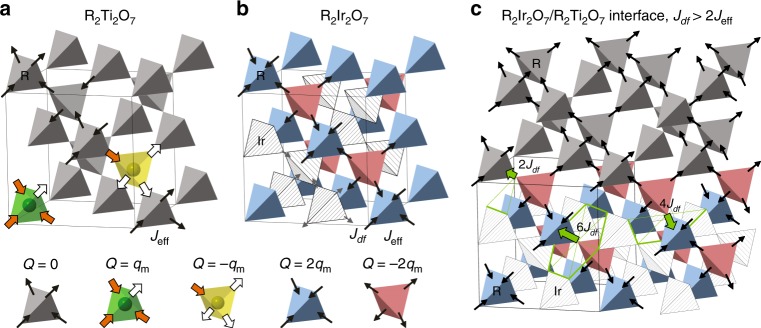
Lattice and spin structure of pyrochlore spin ice, antiferromagnet, and their interface. **a**, **b** Illustrations of lattice and spin structures of a spin ice pyrochlore R_2_Ti_2_O_7_ and an antiferromagnetic pyrochlore R_2_Ir_2_O_7_ respectively, where the nearest neighbor interaction between R^3+^ moments *J*_eff_ and the *d*–*f* interaction between a R^3+^ moment and an Ir^4+^ moment *J*_*df*_ are shown. The arrows represent the local moments. The tetrahedral sites with magnetic charge *Q* = 0 are in gray, *Q* = *q*_m_ in green, *Q* = −*q*_m_ in yellow, *Q* = 2*q*_m_ in blue, and *Q* = −2*q*_m_ in red. **c** Illustration of lattice and spin structure of an R_2_Ir_2_O_7_/R_2_Ti_2_O_7_ interface with *J*_*df*_ >  2 *J*_eff_, where the spin ice layer are forced to adopt a fully polarized state. The complete/truncated Ir^4+^ hexagons are highlighted in green and the complete/reduced local exchange fields experienced by the R^3+^ moments are materialized by green arrows.

Figure 1a,b shows the crystal and spin structures of spin ice R_2_Ti_2_O_7_ and antiferromagnetic R_2_Ir_2_O_7_. In the spin ice, R^3+^ has Ising moments on the order of  ~10 *μ*_*B*_, and obey the “2-in-2-out” ice rules below  ~1K. Spin-flip excitations generate tetrahedral sites with “3-in-1-out/3-out-1-in”, which have been described as quasiparticles akin to magnetic monopoles. In the iridate, the Ir^4+^ forms another tetrahedral network, with an antiferromagnetic “all-in-all-out” (AIAO) order at *T*_*N*_ ~ 130K. The R^3+^ moments in the iridate experience an additional *d*–*f* exchange interaction between R^3+^ and Ir^4+^ moments. Depending on their relative strength to the nearest neighbor interaction *J*_*df*_ /*J*_eff_, R^3+^ moments exhibit an AIAO ordering for *J*_*df*_ /*J*_eff_ > 1, a 3-in-1-out fragmentation configuration for 1/3 < *J*_*df*_ /*J*_eff_ < 1, and remain 2-in-2-out for *J*_*df*_ /*J*_eff_ < 1/3^18^. For simplicity, we first consider the case with AIAO ordering of R^3+^ moments. The typical ordering temperature of R^3+^ moments is a few Kelvins, significantly smaller than ordering temperature of Ir^4+^ moments at  ~130K^19^. As a result, the only moments of interest are the R^3+^, while Ir^4+^ moments are treated as a static antiferromagnetic background. In Fig. 1c we illustrate the crystal and spin structure near a R_2_Ir_2_O_7_/R_2_Ti_2_O_7_ (001) interface. Due to the incomplete coordination of Ir^4+^ nearest neighbors, the exchange fields experienced by the interfacial R^3+^ moments in the R_2_Ir_2_O_7_ and R_2_Ti_2_O_7_ layers are 2/3 and 1/3, respectively, of the strength of those in a bulk R_2_Ir_2_O_7_. When the exchange field is sufficiently strong (i.e., *J*_*df*_ > 2 *J*_eff_), the interface tetrahedra (the topmost red layer) will adopt an AI/AO configuration, forcing the spin ice to adopt a fully polarized state to avoid the formation of monopoles.

**Fig. 2:**
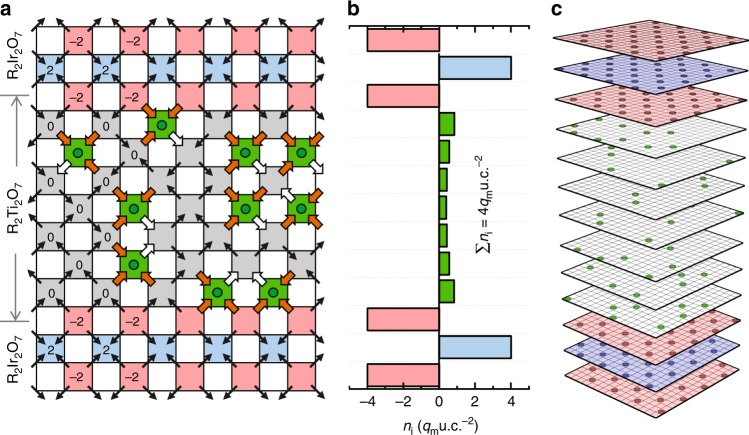
Static snapshot of 2DMG. **a** a 2D illustration of a static snapshot of a 2DMG in a spin ice R_2_Ti_2_O_7_ slab sandwiched between two antiferromagnetic R_2_Ir_2_O_7_ slabs with the boundary moments terminated to be pointing toward the R_2_Ti_2_O_7_ slab. The tetrahedral sites with magnetic charge *Q* = 0 are in gray, *Q* = *q*_m_ in green, *Q* = 2*q*_m_ in blue, and *Q* = −2*q*_m_ in red. **b** A depth profile of the monopole distribution taken from the average of snapshots, and **c** an example of the snapshot of monopole distribution from the ground states in the Monte Carlo results of a R_2_Ir_2_O_7_/R_2_Ti_2_O_7_/R_2_Ir_2_O_7_ sandwich with the nearest neighbor model and a parameter with 6*J*_*df*_/*J*_eff_ = 14.

On the other hand, when the spin ice layer is sandwiched in between two iridate slabs, and polarization directions from both interfaces are opposing, as illustrated in Fig. 2a, the boundary condition forbids the ice rule to be satisfied for every spin ice tetrahedral site. As a result, 3-in-1-out monopole defects with a sheet density of 4 unit cells (u.c.)^−2^ must necessarily exist inside the spin ice.

Unlike in bulk spin ice, where a gas of monopoles and antimonopoles are generated in equal numbers, these monopole defects possess a macroscopic net charge^20^. Also, unlike monopoles in bulk spin ice, which are associated with a spin-flip excitation, the emergence of these monopoles is not accompanied by the creation of antimonopoles in the spin ice, nor defects in R_2_Ir_2_O_7_. In practice, a macroscopic sample will possess random domains where AO (AI) terminations on both interfaces lead to positively (negatively) charged monopoles, whereas AO/AI combinations should not lead to monopoles (see Supplementary Note 9). In antiferromagnetic pyrochlores, the AI/AO domain size can be as large as few tens of microns, as observed using X-ray micro diffraction and microwave impedance microscopy techniques^21,22^, which is sufficiently large such that single domains can be investigated. Moreover, these domains can be trained by cooling down under external fields^21,22^ or via local exchange fields when the iridate is adjacent to a ferromagnetic material, to achieve larger domain sizes. In Fig. 2a, the monopoles are all positively charged in the present boundary condition with ${q}_{m}=(8/\sqrt{3})\mu /a$ where *μ* is the R^3+^ moment and *a* is the pyrochlore lattice constant^3^. As it does not cost energy to flip a spin connecting a 3-in-1-out site and a 2-in-2-out site, the monopoles should be mobile and form a 2DMG. We also have shown that the above picture can be generalized to a single R_2_Ir_2_O_7_/R_2_Ti_2_O_7_ interface, (111)-oriented heterostructures, or even artificial spin ice systems^23^ (see Supplementary Note 13).

### Simulation results of nearest neighbor 2DMGs

To quantitatively explore the physics of this interface, we employ Monte Carlo simulations to investigate the magnetic and thermodynamic properties of the heterostructure including both R_2_Ti_2_O_7_ spin ice^24^ and R_2_Ir_2_O_7_ antiferromagnetic compounds^18^. For simplicity, we initially consider only nearest neighbor interactions:

1 $${\mathcal{H}}={J}_{\text{eff}}\sum _{\langle i,j\rangle }{\sigma }_{i}{\sigma }_{j}-\frac{1}{6}{H}_{\text{loc}}\sum _{\langle i,\alpha \rangle }{\sigma }_{i}{\tilde{\sigma }}_{\alpha }$$

where ${\sigma }_{i},{\tilde{\sigma }}_{\alpha }=\pm\! 1$ are the R^3+^ and the Ir^4+^ Ising pseudo-spins pointing toward or away from a tetrahedron, 〈*i*, *j*〉 represents summations over nearest neighboring sites, *J*_eff_ is the effective nearest neighbor interaction between R^3+^ moments, and *H*_loc_ = 6*J*_*df*_ is a material-dependent tunable parameter that depends on the *d*-*f* exchange interaction between R^3+^ and Ir^4+^ moments. In this model, the altered local environment of the interfacial R^3+^ moments are simply treated by the summation according to the reduced coordination of nearest neighboring Ir^4+^ moments, as shown in Fig. 1c. In Fig. 2c, we show a snapshot of the ground state monopole distribution in an 8-atomic-layer-thick R_2_Ti_2_O_7_ slab sandwiched between two R_2_Ir_2_O_7_ slabs with (001) orientation for *H*_loc_ /*J*_eff_ = 14. In the R_2_Ti_2_O_7_ slab, a gas of singly charged monopoles is evident. Averaging over snapshots gives a cross-sectional monopole density profile as shown in Fig. 2b, showing that the monopoles are denser closer to the R_2_Ir_2_O_7_/R_2_Ti_2_O_7_ interface. Investigations of the thickness dependence of the spin ice slab shows that the 2DMG is confined within 3–4 unit cells of the interface due to entropic considerations (see Supplementary Notes 2 and 5). The integrated sheet density of the entire R_2_Ti_2_O_7_ slab is *n*_2D_ = 4*q*_*m*_u.c.^−2^, independent of the R_2_Ir_2_O_7_ or R_2_Ti_2_O_7_ layer thickness, consistent with the origin of the 2DMG arising from the boundary conditions.

**Fig. 3:**
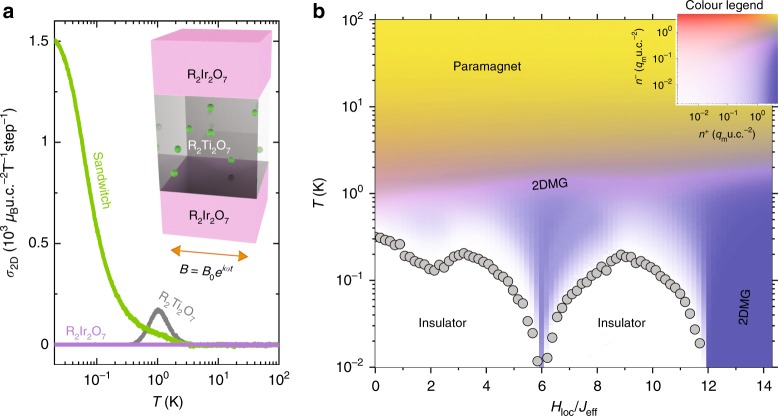
Transport and thermodynamic properties of a R_2_Ir_2_O_7_/R_2_Ti_2_O_7_/R_2_Ir_2_O_7_ (001) sandwich. **a** Idealized monopole A.C. sheet conductivity of the sandwich, whose unit is defined as Bohr magneton per squared pyrochlore unit cell per Tesla per Monte Carlo step, taken at the frequency of 1/20 Monte Carlo steps as a function of temperature with *H*_loc_∕*J*_eff_ = 14 showing a metallic transport behaviour as well as those for a pure iridate slab and a pure spin ice slab as references showing insulating transport behaviours. **b** The *H*_loc_∕*J*_eff_-T phase diagrams of the sandwich showing the monopole sheet magnetic charge density *n*^+^ and the antimonopole sheet density *n*^−^. The above simulations are made with the nearest neighbor model.

To investigate the transport characteristics of the 2DMG, we apply an oscillating longitudinal magnetic field *B*_0_*e*^−*iωt*^ (*B*_0_ = 0.01T and 1/(2*πω*) = 20 Monte Carlo steps) and calculate the sheet conductivity *σ*_2D_ = (*BS*)^−1^*dM* /*dt* ~ *J*_*m*_ /*B* of the sandwich structure, as well as a pure spin ice and a pure iridate slab for baseline comparisons, where *M* is the total magnetization of the system, *S* is the in-plane area of the heterostructure, and *J*_*m*_ is the magnetic charge current, as shown in Fig. 3a. As expected, neither the bulk iridate or spin ice materials exhibit appreciable conductivity at the lowest temperatures. The peak in the conductivity of the bulk spin ice arises from the combination of paramagnetism above 1K and monopole-antimonopole recombination processes below 1K. For the sandwich structure, the conductivity begins to increase around 1K, and monotonically increases with decreasing temperature. While such behavior is reminiscent of a metal, in sharp contrast to its bulk constituents, we emphasize that this system cannot exhibit a finite DC conductivity in steady state.

In Fig. 3b, we show the total sheet density of monopoles *n*^+^ and antimonopoles *n*^−^ as a phase diagram of temperature and *H*_loc_ /*J*_eff_. Above the spin ice freezing temperature of a few Kelvins, the system is paramagnetic and dominated by thermal fluctuations. For 0 < *H*_loc_ /*J*_eff_ < 12, a singly charged 2DMG always exists within a finite temperature window, due to the non-zero flux of the exchange field that flows toward/away from the spin ice. As the temperature is lowered toward the ground state, the system exhibits different phases for various *H*_loc_ /*J*_eff_ values. For *H*_loc_ /*J*_eff_ > 12, a metallic 2DMG persists to the lowest temperatures, which is the case as shown in Fig. 2. For 6 < *H*_loc_ /*J*_eff_ < 12 and 2 < *H*_loc_ /*J*_eff_ < 6 regions, where the iridate adopts AIAO and fragmented phases respectively, the boundary tetrahedral layers have pinned, fragmented charges due to the insufficient interfacial exchange fields. As a result, a 2DMG exists at finite temperatures for these *H*_loc_ /*J*_eff_ values, but is not the ground state. At *H*_loc_ /*J*_eff_ = 6, the iridate is on the phase boundary between the AIAO and fragmentation phases, and the 2DMG state arises from the resulting spin fluctuations. Nevertheless, a charged 2DMG exists over all values of *H*_loc_ /*J*_eff_ in the phase diagram, and thus should be experimentally accessible in real material systems.

**Fig. 4:**
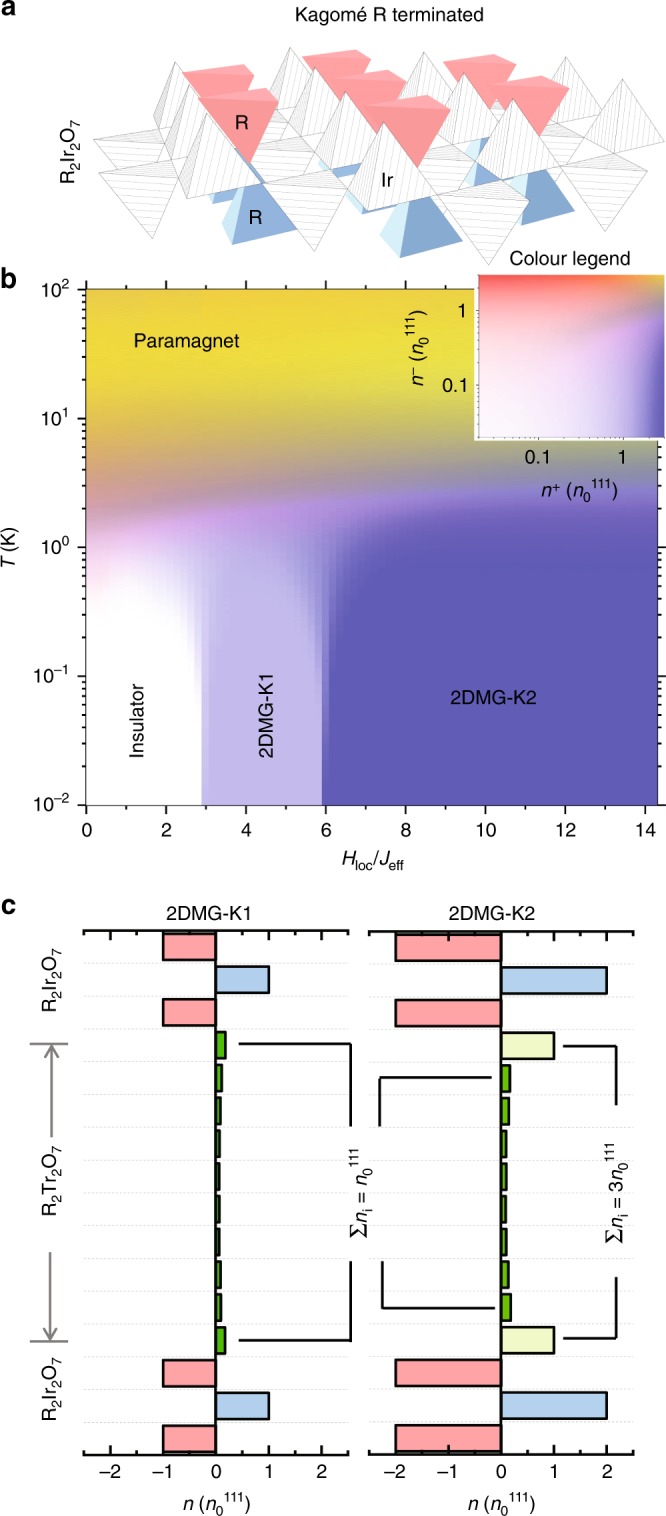
2DMG with (111) orientation. **a** Illustration of the lattice structure of the R_2_Ir_2_O_7_/R_2_Ti_2_O_7_ (111) interface. **b** Monopole (antimonopole) sheet density *n*^+^(*n*^−^) of the sandwich. Two distinct 2DMG phases are observed, as characterized by different monopole densities in the spin ice slab. **c** Monopole distribution profiles of both 2DMG phases at the ground state. The mobile 2DMG layers are labeled by green bars, whereas the immobile monopole layers are labeled by light green bars. The above simulations are made in R_2_Ir_2_O_7_/R_2_Ti_2_O_7_/R_2_Ir_2_O_7_ (111) with the nearest neighbor model.

We have also investigated the 2DMG in R_2_Ir_2_O_7_/R_2_Ti_2_O_7_/R_2_Ir_2_O_7_ (111) sandwiches with a Kagomé R^3+^ termination on the iridate layer, as shown in Fig. 4a. In Fig. 4b we plot the *H*_loc_ /*J*_eff_-*T* phase diagram of the sheet density of monopoles and antimonopoles. This phase diagram is qualitatively similar to that of a (001)-oriented heterostructure, but here the 2DMG ground state is even more robust. In the region with 3 < *H*_loc_ /*J*_eff_ < 6, a 2DMG (which we label as 2DMG-K1) exists with a sheet density of ${n}_{0}^{111}$ (where ${n}_{0}^{111}=4{q}_{m}/\sqrt{3}$*a*^−2^ is a sheet density when 3-in-1out monopoles fully occupy a single tetrahedra layer along the (111) direction), and covers the experimentally determined value of *H*_loc_ /*J*_eff_ = 4.5 for Ho_2_Ir_2_O_7_^18^. For *H*_loc_ /*J*_eff_ > 6, the total sheet density increases to $3{n}_{0}^{111}$, (labeled as 2DMG-K2), where the additional $2{n}_{0}^{111}$ arises from immobile monopoles pinned to the interfacial layers, as shown in Fig. 4c. We have also calculated the (111) sandwich structure with the triangular R^3+^  termination, and found that its behavior is qualitatively similar to the Kagomé termination.

**Fig. 5:**
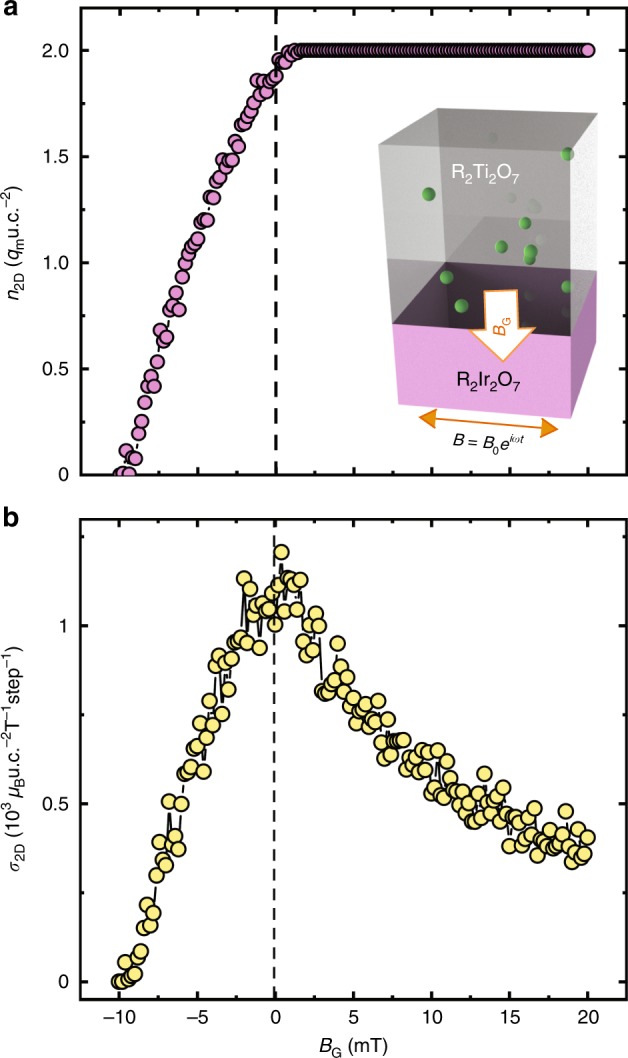
Magnetic gating effects on 2DMGs. **a** Monopole sheet density *n*_2D_ and **b** sheet conductivity *σ*_2D_ as a function of a vertical magnetic gating field *B*_G_. We choose to study a positively charged 2DMG with all-out termination on the boundary. The definition of positive *B*_G_ is shown in the inset of panel **a**. The Monte Carlo simulation is performed at 0.1 K on an R_2_Ir_2_O_7_/R_2_Ti_2_O_7_ (001) single interface with the nearest neighbor model and *H*_loc_∕*J*_eff_ = 14.

In Fig. 5, we demonstrate that the sheet density of the 2DMG can be controlled by an external, out-of-plane magnetic field. Here, we choose a single interface, because an out-of-plane field will not deplete or accumulate the 2DMG in the sandwich configuration shown in Fig. 2, but rather preferentially move monopoles towards one or the other interface. Although the 2DMG is not the ground state for a single interface, it can be thermally excited at arbitrarily low temperatures which scale inversely with the thickness of the spin ice layer (e.g.,  ~700 nK for a 1 mm thick layer, the thickness of a typical substrate or bulk crystal). Figure 5a shows the monopole sheet density as a function of magnetic field *B*_G_ (similar to a gate voltage in a field effect transistor), with positive direction defined as pointing from the iridate to the spin ice. With a negative field, the monopoles are driven away from the interface and pushed into the bulk of the iridate, and the 2DMG can then be fully depleted at a *B*_G_ of 10 mT, while with a positive field, the monopoles are driven toward the interface, and saturate at a density of 2*q*_*m*_u.c.^−2^, since the maximum sheet density is determined by the boundary conditions. Figure 5b shows the monopole sheet conductivity *σ*_2D_ as a function of *B*_G_. In negative *B*_G_, *σ*_2D_ decreases toward zero as the 2DMG is depleted, whereas at positive bias *σ*_2D_ also decreases since the the monopoles are pushed toward the boundary, thereby reducing the number of empty sites into which a monopole can hop.

### Simulation results of dipolar 2DMGs

Having demonstrated the existence and properties of a 2DMG in the simplest nearest neighbor-only model, we now explore the effects of the long-range dipolar interaction between R^3+^ − R^3+^, which should act as an effective Coulomb interaction between monopoles. We find that the existence of the charged 2DMG is robust against dipolar interactions. We now add these additional non-nearest neighboring terms into the Hamiltonian:

2 $${\mathcal{H}}\, =	 \; {J}_{\text{eff}}\sum _{\langle i,j\rangle }{\sigma }_{i}{\sigma }_{j}-\frac{1}{6}{H}_{loc}\sum _{\langle i,\alpha \rangle }{\sigma }_{i}{\tilde{\sigma }}_{\alpha }\\ 	 +\frac{{\mu }_{0}{\mu }^{2}}{4\pi }\sum _{{(i,j)}^{\prime}}\left[\frac{{{\bf{e}}}_{i}\cdot {{\bf{e}}}_{j}}{| {{\bf{r}}}_{ij}{| }^{3}}-\frac{3({{\bf{e}}}_{i}\cdot {{\bf{r}}}_{ij})({{\bf{e}}}_{j}\cdot {{\bf{r}}}_{ij})}{| {{\bf{r}}}_{ij}{| }^{5}}\right]{\sigma }_{i}{\sigma }_{j}$$

where ${(i,j)}^{\prime}$ represents summations over non-nearest neighboring sites, **e**_*i*_ is a unit vector along the positive direction of an Ising pseudo-spin *σ*_*i*_.

**Fig. 6:**
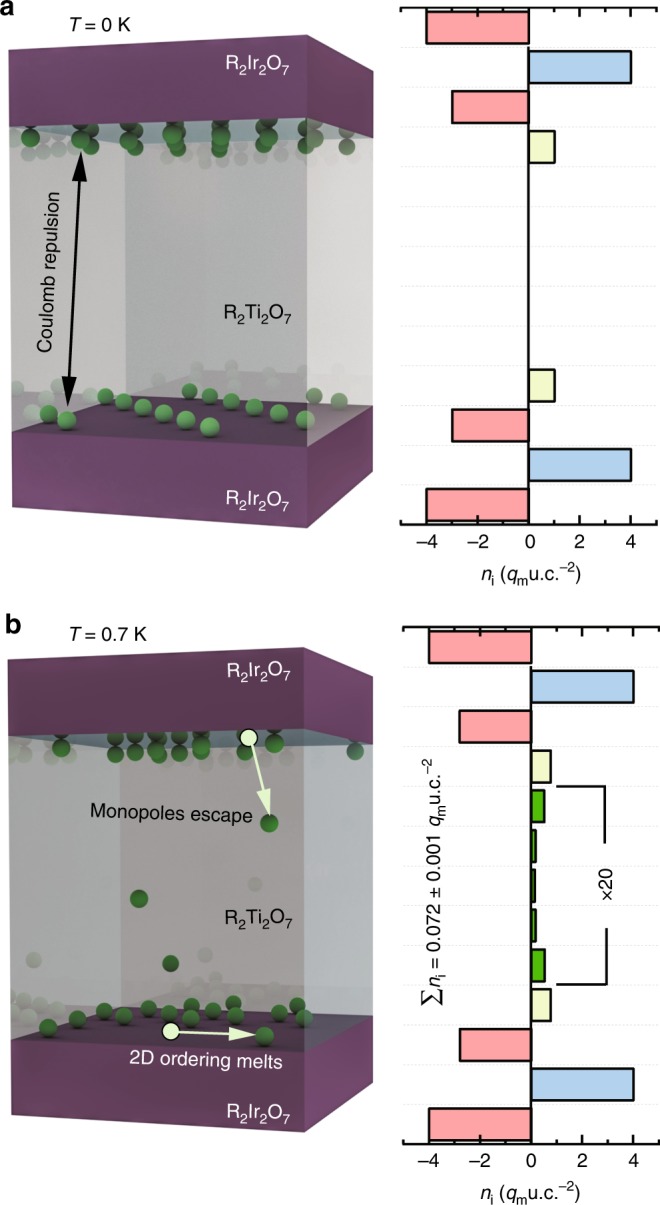
Effects of long-ranged interaction on 2DMGs. Depth profiles and 3D illustrations of monopole distributions of a R_2_Ir_2_O_7_/R_2_Ti_2_O_7_/R_2_Ir_2_O_7_ sandwich with dipolar interactions turned on. **a** In the ground state, the monopoles form a 2D stripe ordering at the boundary layers. **b** At 0.7 K, the 2D order melts and some monopoles enter the interior of the spin ice layers. The sheet density of escaped monopoles is shown. The Monte Carlo simulation is performed in a R_2_Ir_2_O_7_/R_2_Ti_2_O_7_/R_2_Ir_2_O_7_ (001) sandwich and *H*_loc_∕*J*_eff_ = 10.

 Figure 6a shows the ground state snapshot of the monopole distribution of a trilayer of a spin ice slab (eight atomic layers) sandwiched in between two iridate slabs for *H*_loc_ /*J*_eff_ = 10, taking into account dipolar interactions. Due to this effective Coulomb repulsion, monopoles are pushed to the boundary layer and form a two-dimensional stripe-like ordering with ***q*** = (*π* /2, *π* /2), reminiscent of the charge-stripe ordering observed in complex oxides such as cuprates with strong electron correlations^25^. Upon warming, this ordered state melts and some monopoles overcome their Coulomb repulsion and enter the interior of the spin ice layer, forming a 2DMG similar to the simpler nearest-neighbor-only case illustrated in Fig. 6b. The sheet density of the interacting 2DMG is considerably smaller than that of the non-interacting, nearest-neighbor-only 2DMG, since due to the long-range interactions, many of the monopoles remain confined to the boundary layer. Although quantitative details about the density and distribution are affected by the long-ranged interactions, our calculations nevertheless show that the existence of the 2DMG is indeed robust against Coulomb interactions.

## Discussion

These proposed heterostructures can be realized using modern thin film growth techniques such as molecular beam epitaxy or pulsed laser deposition, owing to the stable +4 valence of Ir and Ti, as demonstrated by the recent successful growth of the spin ice and the iridate thin films^26–29^. While the lattice mismatch between bulk spin ice and iridate compounds is comparatively small (1%), even a modest epitaxial strain can lift frustration in the spin ice layer^26^. To avoid this complication, the heterostructure can be synthesized on a substrate which is directly lattice matched to the spin ice material (e.g. a pyrochlore titanate single crystal), since the antiferromagnetism in the iridate layer is likely more robust against small anisotropies. Finally, the magnetization signal from the motion of the 2DMG should, in principle, be detectable by conventional superconducting quantum interference device magnetometers (10^−8^ emu sensitivity), since the sheet density of monopoles is estimated to be  ~4 × 10^14^ cm^−2^ or  ~7 × 10^12^ cm^−2^ for the nearest neighbor only and dipolar calculations, respectively. Assuming a detection area on the order of a few square millimeters, the hopping of the dipolar 2DMG should yield a changing magnetization signal of ~10^−7^ emu.

The proposed 2DMG should enable possibilities in the study of emergent magnetic monopoles not possible in bulk materials. Its active magnetic charge degree of freedom should allow for the isolation of monopoles or antimonopoles^30^, while the lowered dimensionality of the system should facilitate the fabrication of transistor-like geometries where the density of monopoles can be actively controlled. This will allow one to address outstanding questions including whether emergent monopoles carry an electric dipole akin to the spin of an electron^30^, whether they experience Lorentz force in electric fields and exhibit an analogous Hall effect, and their energy-momentum dispersion relationship.

## Methods

### Monte Carlo simulations

The Monte Carlo simulations have been performed based on the Ising model of the pyrochlores, with *σ*_*i*_ = ±1 being the Ising pseudo-spin representation of a R^3+^ moment pointing toward or away from a given tetrahedron. The value of *σ*_*i*_ is then simulated according to the spin model and the Monte Carlo method with the standard Metropolis algorithm^18,24^. For the nearest neighbor-only model, a single spin-flip algorithm was combined with a worm-loop algorithm to prevent the system from being trapped in a local minimum at low temperatures. For the long-ranged interactions, a parallel algorithm was also used. The worm-loop algorithm developed for the pyrochlore heterostructures was based on the loop algorithm used in previous works^18,24,31,32^ (see Supplementary Note 4). For R_2_Ir_2_O_7_/R_2_Ti_2_O_7_/R_2_Ir_2_O_7_ heterostructures, the simulations were performed on 16 × *I*^2^ × *K* lattice sites with periodic boundary conditions, where *I* = 4 and *K* = 12. For R_2_Ir_2_O_7_/R_2_Ti_2_O_7_ heterostructures, the simulations were performed on 16 × *I*^2^ × *K* lattice sites with periodic boundary conditions along in-plane directions, where *I* = 4 and *K* = 30. We have increased the in-plane model size from *I* = 4 to *I* = 10 and did not see difference in 2DMG properties. We have also varied the spin ice slab thickness, and discovered the natural confinement depth of the 2DMG. In total, 10^6^ hybrid Monte Carlo steps were used for the thermalization, including at least 10^7^ single spin-flip steps.

## Supplementary information

Supplementary Information

Peer Review File

## Data Availability

All relevant data are available from the authors upon reasonable requests.

## Code availability

All relevant codes are available from the authors upon reasonable requests.
