# Supplementary material for: Two-dimensional magnetic monopole gas in an oxide heterostructure
Source: Nat Commun. 2020 Mar 12;11:1341. doi: 10.1038/s41467-020-15213-z (PMC7067881; doi:10.1038/s41467-020-15213-z)
Supplement: Supplementary file 1 — Supplementary Information [file 41467_2020_15213_MOESM1_ESM.pdf]

Supplementary Information

Two-dimensional magnetic monopole gas in an oxide heterostructure

Miao *et al.*

Supplementary Note 1: The comparison between the two dimensional monopole gas (2DMG) at the antiferromagnet (AFM)/spin ice interface and the two dimensional electron gas (2DEG) at the $LaAlO_3$ (LAO)/$SrTiO_3$ (STO) interface

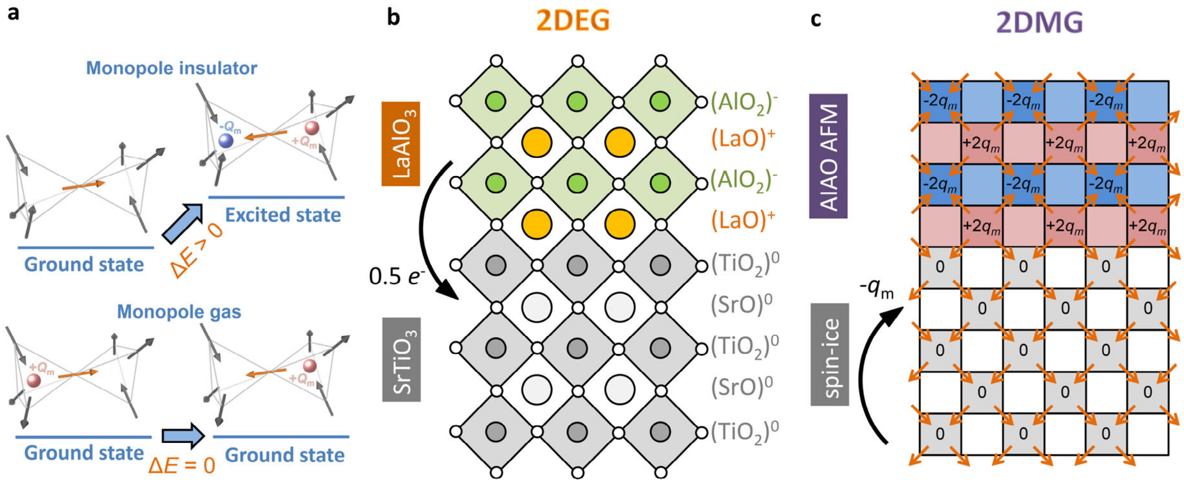

Supplementary Figure 1 | Illustration of the 2DMG at the AFM/spin-ice interface and the 2DEG at the LAO/STO interface a. Ground states and excitation states for a monopole insulator and a free monopole gas. Illustrations of **b**. a 2DEG at the interface between LAO and STO and **c**. a 2DMG at the interface between an all-in-all-out (AIAO) AFM and a spin ice.

The comparison between a monopole insulator and a monopole gas is shown Supplementary Figure 1**a**. The spin ice is a monopole insulator, because it requires a finite energy to overcome the 2-in-2out rule for two tetrahedral sites to flip a spin in between them. In contrast, the hopping of a free monopole does not cost energy, as the hopping process does not create additional monopoles. To realize such a state as the ground state, requires unequal numbers of monopoles and antimonopoles.

Supplementary Figure 1**b** and **c** shows the atomic structure of the interface between $LaAlO_3$ and $SrTiO_3$, and the spin structure of the interface between the AFM and the spin ice. The two systems resemble in terms of polar discontinuity. However, the mechanisms of the interface gas are different: for the 2DEG at LAO/STO interface, the mechanism is polar catastrophe and the 2DEG requires a minimum LAO thickness of 3 u.c.. For the 2DMG at the AFM/spin ice interface, on the other hand, the mechanism is a combination of a time-reversal symmetry breaking boundary condition plus the minimization of the free energy. (See Supplementary Note 2 for detailed discussions).

Supplementary Note 2: Mechanism of natural confinement of the 2DMG and the mechanism of a finite-temperature 2DMG state in an $R_2Ir_2O_7$ (RIO)/$R_2Ti_2O_7$ (RTO) single interface

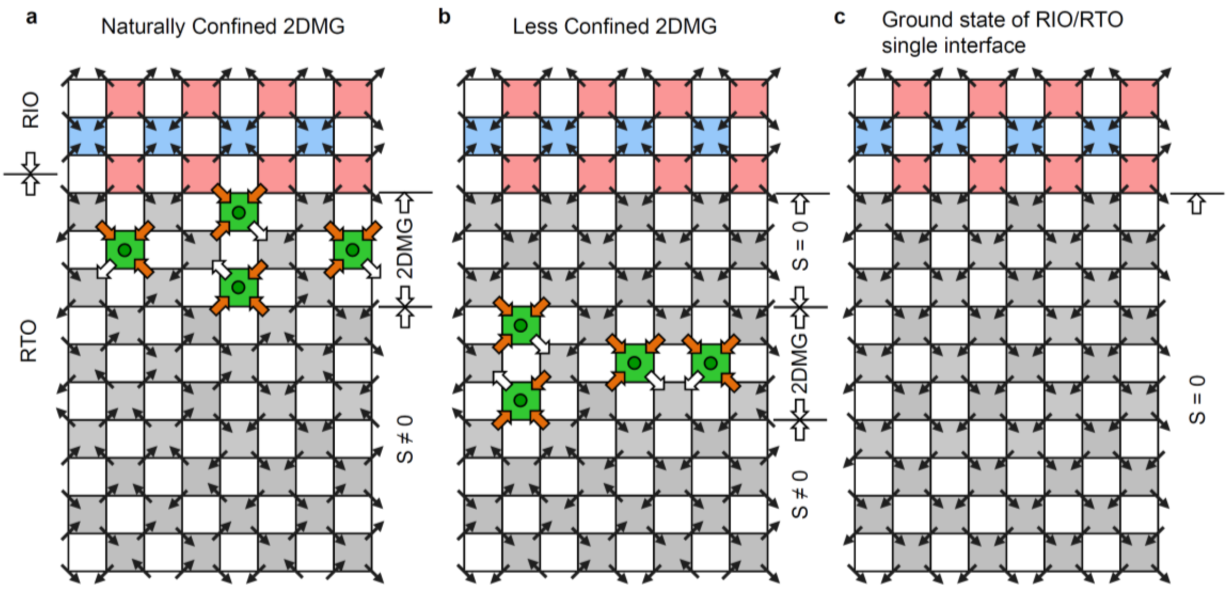

Supplementary Figure 2 | Illustration of entropies of the RIO/RTO single interface of different configurations. a. a 2DMG naturally confined near the interface, restoring the frustrated Coulomb phase in the rest of the RTO slab. **b**. a less confined 2DMG, creating a fully polarized region between the 2DMG and the interface. **c**. the true ground state of a RIO/RTO, where the entire RTO slab has zero entropy.

Consider two spin configurations of a RIO/RTO interface, as shown in Supplementary Figure 2 **a** and **b**, where the configuration in Supplementary Figure 2**b** is less confined. Although both configurations are energetically equivalent, the less confined state in Supplementary Figure 2**b** has lower entropy, as there is a fully polarized, zero entropy region between the interface and the 2DMG. Therefore the 2DMG is naturally confined due to entropic reasons.

We also note that the ground state of a single RIO/RTO interface is as shown in Supplementary Figure 2**c**, absent of 2DMG but zero entropy. However, a 2DMG state can be excited at a finite temperature to minimize the free energy U-TS, where the first term is 2DMG forming energy and the second term includes the spin ice slab zero-point entropy. Since the second term is proportional to RTO slab thickness, and the first term is a constant, the temperature needed to excite a 2DMG state should be inversely proportional to the RTO slab thickness. For example, 2DMG state will occur above 7 mK with RTO (R = Dy) thickness of 100 nm, above 700 nK with thickness of 1mm, and above any infinitesimal temperature in theory when the RTO slab is infinite.

Supplementary Note 3: Interactions among R^{3+} and Ir^{4+} moments in $R_2Ir_2O_7$

A perfect candidate of the AFM material is $R_2Ir_2O_7$ (RIO). In RIO, Ir^{4+} and R^{3+} form two identical sets of corner shared tetrahedral networks. Due to the strong spin-orbit coupling, both moments of Ir^{4+} and R^{3+} are both locked to a direction that points either toward or away from the adjacent tetrahedral center [1]. In this system, there are *d-d* exchange interactions between Ir^{4+} moments, *d-f* exchange interactions between Ir^{4+} and R^{3+} moments, and *f-f* exchange and dipolar interactions between R^{3+} moments:

$$\mathcal{H} = J_{dd}\mu_{\mathrm{Ir}}^2 \sum_{<i_{\mathrm{Ir}},j_{\mathrm{Ir}},NN>} \boldsymbol{S}_{i_{\mathrm{Ir}}}\cdot\boldsymbol{S}_{j_{\mathrm{Ir}}} + J_{df}\mu_{\mathrm{R}}\mu_{\mathrm{Ir}} \sum_{<i_{\mathrm{Ir}},j_R,NN>} \boldsymbol{S}_{i_{\mathrm{Ir}}}\cdot\boldsymbol{S}_{j_R} + J_{ff}\mu_R^2 \sum_{<i_R,j_R,NN>} \boldsymbol{S}_{i_R}\cdot\boldsymbol{S}_{j_R}$$

$$+\frac{\mu_0\mu_R^2}{4\pi}\sum_{(i_R,j_R)}\left[\frac{\boldsymbol{S}_{i_R}\cdot\boldsymbol{S}_{j_R}}{|\boldsymbol{r}_{i_R,j_R}|^3} - \frac{3(\boldsymbol{S}_{i_R}\cdot\boldsymbol{r}_{i_R,j_R})(\boldsymbol{S}_{j_R}\cdot\boldsymbol{r}_{i_R,j_R})}{|\boldsymbol{r}_{i_R,j_R}|^5}\right] \quad (1)$$

where $\mu_{\mathrm{Ir}} \sim 1\mu_{\mathrm{B}}$ is the local moment for Ir^{4+} ions and $\mu_{\mathrm{R}} \sim 10\mu_{\mathrm{B}}$ is the local moment for R^{3+} ions, μ_{B} is the Bohr magneton, $\boldsymbol{S}$ is a unit vector along the local moment direction, J_{dd}, J_{df}, J_{ff} are the coefficients of *d-d*, *d-f* and *f-f* nearest-neighbor (NN) exchange interactions respectively. First, because *d-d* exchange interactions are so much stronger than all other interactions, the Ir^{4+} networks orders with AIAO configuration at ~ 130 K for $Ho_2Ir_2O_7$ and ~133 K for $Dy_2Ir_2O_7$, respectively, which is two orders of magnitude higher than the ordering temperature of R^{3+} moments of ~ 1 K [2]. Therefore, around the temperature range where R^{3+} moments start to order, Ir^{4+} moments are considered to be fixed. When Ir^{4+} moments order, they can form an AIAO or AOAI domain, which is a time reversal copy to each other [3]. The domain size is about sub μm large. Because the *d-d* exchange interactions is short range and so much stronger any all other interactions in the system, for RIO/RTO/RIO heterostructure the Ir^{4+} domain forming in the top RIO layer and the bottom RIO layer should be

independent, yielding to four possible domains. Second, the *f-f* term and the dipolar term are almost identical to those in spin ice, owing to the similar lattice constant between RIO and RTO. Then the remaining term is the *d-f* term. It can be considered as the staggered magnetic field acting on the R^{3+} ions [4]:

$$\mathcal{H}_{df} = -\mu_R \sum_{i_R} \boldsymbol{H}_{\text{loc}}(i_R) \cdot \boldsymbol{S}_{i_R}, \text{where } \boldsymbol{H}_{\text{loc}}(i_R) = -J_{df}\mu_{\text{Ir}} \sum_{<j_{Ir},NN>} \boldsymbol{S}_{j_{Ir}} \quad (2)$$

Supplementary Note 4: Worm-loop algorithm

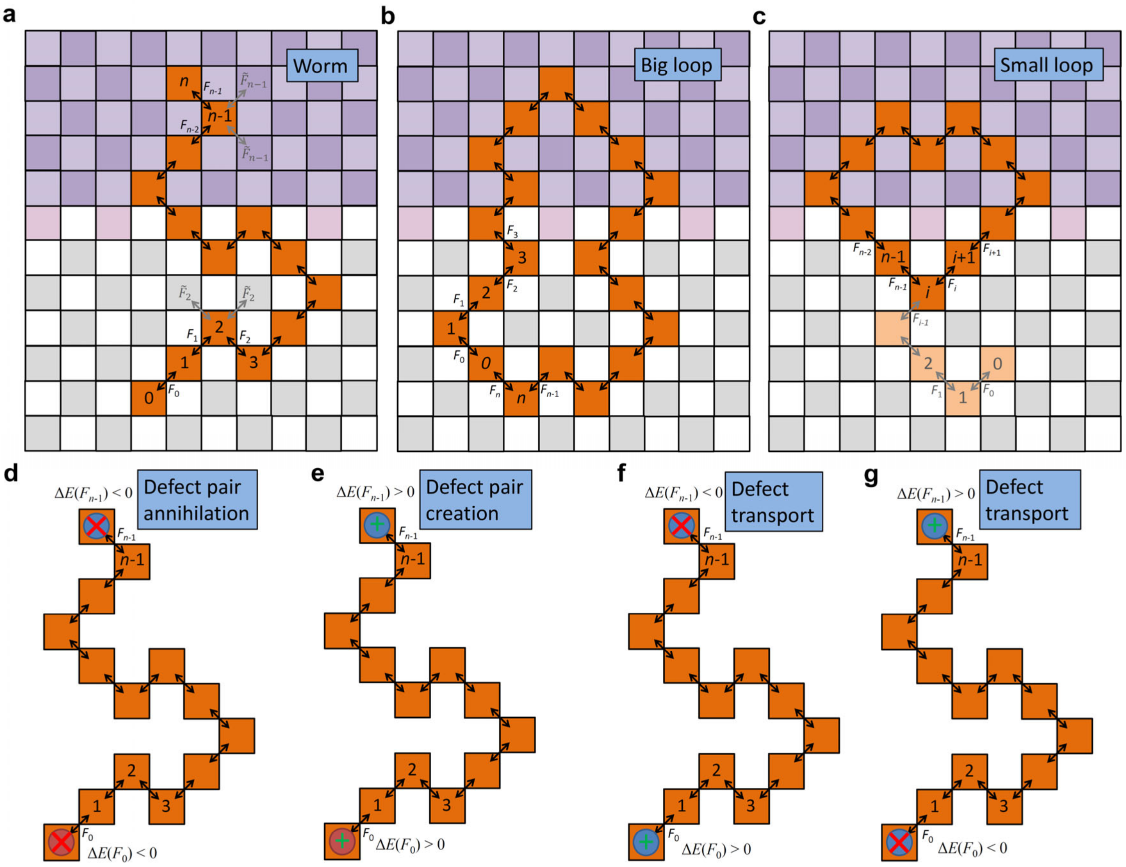

Supplementary Figure 3 | An illustration of the worm-loop algorithm. Illustrations of **a.** a worm path, **b.** a big loop and **c.** a small loop with spin flips, where the adopted spin flips F_i and the abandoned spin flips $\tilde{F}_i$ have been labeled. **d.-g.** Illustrations of four possible scenarios associated with a worm path, i.e. the annihilation of a pair of defects, the creation of a pair of defects, and the transport of a single defect.

To prevent the system from being stuck at a local minimum in the Monte Carlo simulation for pure RTO and RIO materials, people developed an algorithm called loop algorithm [1,5-7]. The spirit of the loop algorithm is to flip multiple spin at a time while the system is stuck during the single-spin flip (SSF) process. The first step is to tentatively flip a spin in regardless whether it is energy favorable or not. Then tentatively flip one of the next

neighboring spin to restore the local rules (2-in-2-out for spin ice, 3-in-1-out for fragmentation phase), until a loop of spin is flipped. However, the loop algorithm does not create, annihilate, or transport any monopole excitations in RTO or local defects in RIO. Since the local rules should be satisfied everywhere in pure RTO and RIO materials at the ground state, there are no necessities for the loop algorithm to deal with the monopoles or local defects.

To deal with the monopoles at the ground state in our RIO/RTO and RIO/RTO/RIO heterostructures, we developed a new algorithm called "worm-loop" algorithm (WLA) based on the existing loop algorithm, as shown in Supplementary Figure 3**a**-**c**. The first step is also to tentatively flip a spin F_0 in regardless whether it is energy favorable or not. But the choice of the next nearest neighboring spin is not based on any local rules. Rather, it is based on which spin is the most energy favorable. In this case, a chain of spin is flipped. The chain can be either not closed like a worm as shown in Supplementary Figure 3**a**, or closed like loop as shown in Supplementary Figure 3**b** and **c**. The loop cases are similar to the existing loop algorithm. The worm case however, involved the annihilation, creation, or transport of a pair of monopoles or defects, as shown in Supplementary Figure 3**d**-**g**, which is critical for the heterostructures. Finally, the worms or loops will give a total energy change dE. Just like the metropolis algorithm, we accept the cases with d$E \leq 0$, and accept the cases with d$E > 0$ with the probability of exp (-d$E/k_B T$).

Supplementary Note 5: Natural confinement depth of the 2DMG in NN-only model.

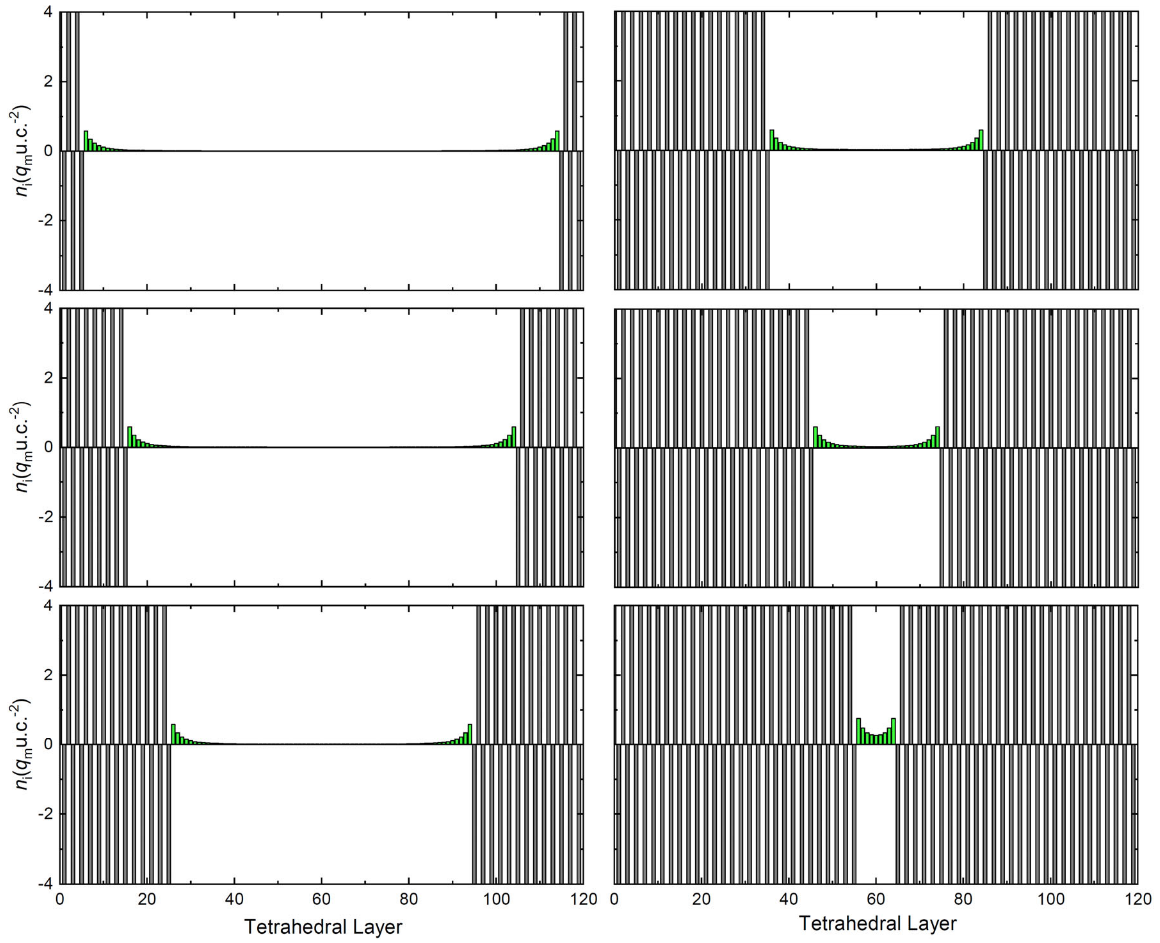

Supplementary Figure 4 | Natural confinement depth of the 2DMG in NN-only model. Monte Carlo simulated monopole distribution of RIO/RTO/RIO (001) heterostructures at ground state with $H_{\mathrm{loc}}/J_{\mathrm{eff}} = 14.2$, with varying RTO thickness, but a constant total thickness. The regions in green are 2DMGs and the regions in grey have AIAO configurations.

In searching for the possible natural confinement of the 2DMG, we have investigated a RIO/RTO/RIO system, with varying the number of RTO layers d_{RTO} under the NN-model, as shown in Supplementary Figure 4. We found that 90% of theses monopoles lives within 15 layers near both RIO/RTO boundary.

Supplementary Note 6: 2DMG mechanism at ground state with various H_{loc}/J_{eff} values

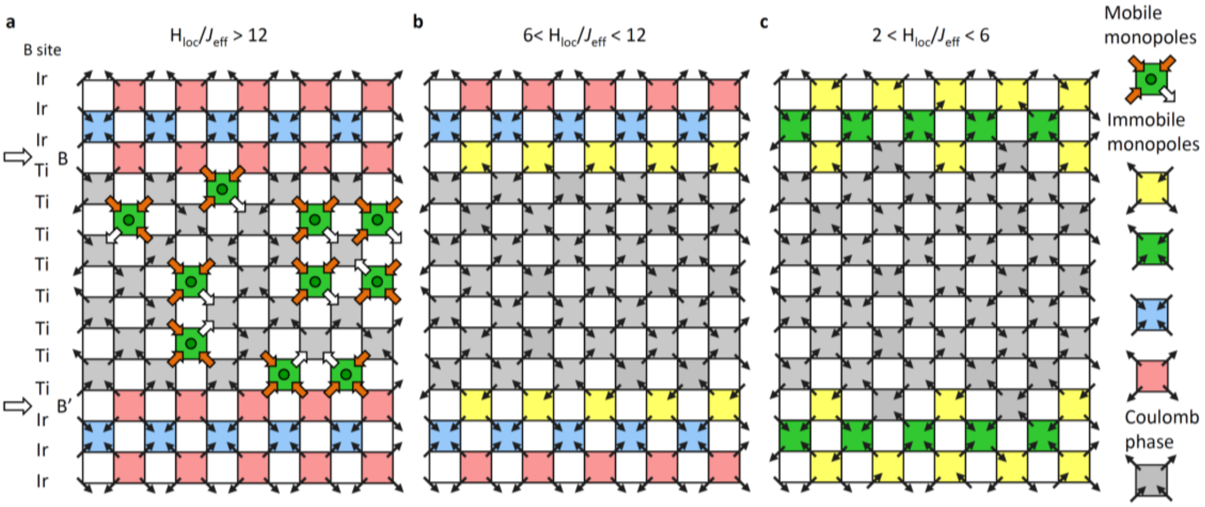

Supplementary Figure 5 | 2DMG mechanism at various H_{loc}/J_{eff} values Simplified 2D illustration of monopole distribution in a RIO/RTO/RIO (001) heterostructure at the ground state, with **a.** H_{loc}/J_{eff} > 12, **b**. 6 < H_{loc}/J_{eff} < 12, and **c**. 2 < H_{loc}/J_{eff} < 6. The RIO/RTO interfaces are labeled by two arrows on the left, and the tetrahedral layers at the interfaces are labeled as B and B'. The B site atoms at each atomic layer are also labeled. Only in the case of H_{loc}/J_{eff} > 12, a mobile 2DMG is expected.

When H_{loc}/J_{eff} is between 6 and 12, RIO interior is still AIAO, but the interfacial tetrahedral layer (B and B') is fragmentation phase. The fragmented monopoles in B and B' cannot leave the layer freely at ground state, as they are pinned by the local molecular field with strength of 1/3H_{loc}. Similarly when H_{loc}/J_{eff} is between 2 and 6, RIO interior is fragmentation phase, whereas charge density in B and B' layers is half of that in RIO interior layers, leading to the absent of 2DMG at the ground state. When H_{loc}/J_{eff} is between 0 and 2 (not shown here), all sites of the entire heterostructure should adopt the 2I2O configuration.

Supplementary Note 7: Phase diagram of an RIO/RTO/RIO heterostructures with NN model

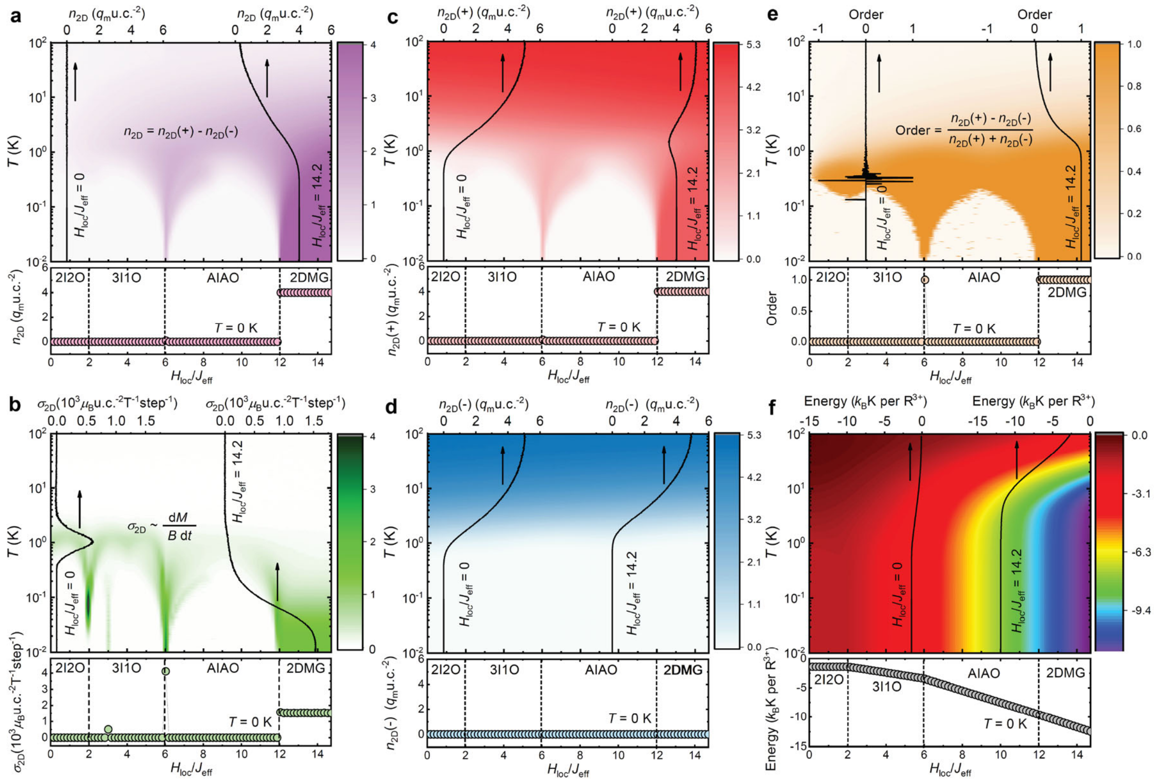

Supplementary Figure 6 | Monte Carlo simulated H_{loc}-T phase diagrams of a RIO/RTO/RIO (001) trilayer. H_{loc}-T phasediagrames of **a.** the sheet density of the net monopoles within RTO layer n_{2D}, overlaid with $n_{2D}(T)$ at $H_{loc}/J_{eff} = 0$ and 14.2. The lower panel is $n_{2D}(H_{loc}/J_{eff})$ function at the ground state. **b.** the sheet monopole conductivity σ_{2D} measured under an AC field with a frequency of 0.05/step and a strength of 0.01 T, overlaid with $\sigma_{2D}(T)$. The lower panel is $\sigma_{2D}(H_{loc}/J_{eff})$ function. The sheet density of **c.** monopoles $n_{2D}(+)$ and **d.** antimonopoles $n_{2D}(-)$ within RTO layer, overlaid with $n_{2D}(+,T)$ and $n_{2D}(-,T)$ functions The lower panels are $n_{2D}(+, H_{loc}/J_{eff})$ and $n_{2D}(-, H_{loc}/J_{eff})$ function, respectively. **e.** Charge polarization ordering parameter as defined as $p_m = [n_{2D}(+) - n_{2D}(-)]/[n_{2D}(+) + n_{2D}(-)]$, overlaid with $p_m(T)$. The lower panel is $p_m(H_{loc}/J_{eff})$ function. **f.** The energy E per R^{3+} local moment overlaid with $E(T)$. The lower panel is $E(H_{loc}/J_{eff})$ function.

In Supplementary Figure 6, we plot The $H_{\mathrm{loc}}/J_{\mathrm{eff}}-T$ phase diagrams of various physical properties including the monopole and antimonopole sheet density $n_{2\mathrm{D,m}}$ and $n_{2\mathrm{D,am}}$, net sheet density $n_{2\mathrm{D}}$, an order parameter of charge polarization of monopole p_{m} defined as $[n_{2\mathrm{D}}(+) - n_{2\mathrm{D}}(-)]/[n_{2\mathrm{D}}(+) + n_{2\mathrm{D}}(-)]$, sheet conductivity $\sigma_{2\mathrm{D}}$, as well as the total energy E for a RIO/RTO/RIO system.

When the temperature is larger than ~ 1K, the system is in a paramagnetic phase, as characterized by a small sheet conductivity and large sheet densities of monopoles and antimonopoles due to thermal fluctuations. Interestingly, the population of monopoles and antimonopoles are not equal already in this regime. To see this, the monopole charge polarization has a non-zero value.

Upon cooling down to temperature roughly between 1 K and 0.1 K, the system enters the 2DMG phase for all the $H_{\mathrm{loc}}/J_{\mathrm{eff}}$ values. In this regime the antimonopoles are annihilated in the RTO layers due to the establishment of 2-in-2-out rule. However, positively charged monopoles still remain in the RTO layers, due to the boundary condition. $p_{\mathrm{m}} = 1$ in this regime, indicating that the 2DMG is made by monopoles with a single sign of charge. Also, the 2DMG start to contribute the sheet conductivity, which has an increased value in this regime.

Upon further cooling down to the ground state, the system will end up differently under different $H_{\mathrm{loc}}/J_{\mathrm{eff}}$ values. For $H_{\mathrm{loc}}/J_{\mathrm{eff}} > 12$, the system enters a metallic 2DMG regime. The 2DMG is still made by purely positively charged monopoles, whose sheet density increased

to large value of 4 q_{m}u.c.$^{-2}$. The transport properties are also metallic. For $H_{\mathrm{loc}}/J_{\mathrm{eff}} < 12$, the system turn insulating again, as all the monopoles in the 2DMG will be absorbed to the interface layers (see Supplementary Figure 5 and 7).

The non-zero monopole conductivity at $H_{\mathrm{loc}}/J_{\mathrm{eff}} = 3$ is observed at ground state for the following reason: the moments at the boundary layers of iridate are experiencing a local field of 2/3 H_{loc}, as shown in Fig. 1**c** in the main article. Similar to the 2I2O/fragmented phase boundary in iridate bulk with $H_{\mathrm{loc}}/J_{\mathrm{eff}} = 2$, these spins can fluctuate without costing energy, generating an AC susceptibility/conductivity.

Supplementary Note 8: Monopole distributions of an RIO/RTO/RIO heterostructures at various H_{loc}/J_{eff} and T values with NN model.

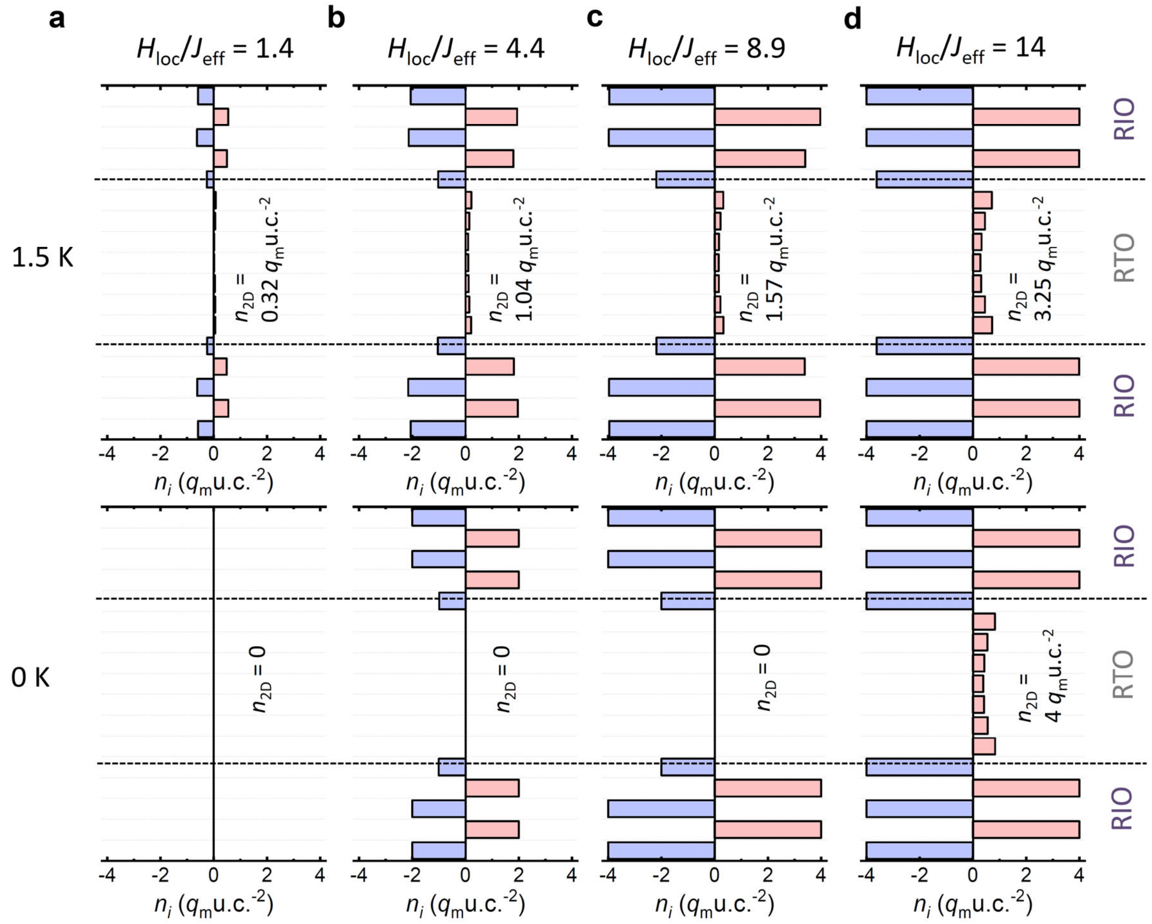

Supplementary Figure 7 | Monopole distribution of an RIO/RTO/RIO heterostructure simulated with NN model. Monopole density distribution as a function of tetrahedral layer positions at the ground state and 1.5 K, for a system with **a.** $H_{loc}/J_{eff} = 1.4$, **b.** $H_{loc}/J_{eff} = 4.4$, **c.** $H_{loc}/J_{eff} = 8.9$ and **d.** $H_{loc}/J_{eff} = 14$ respectively.

Supplementary Figure 7 shows the monopole distribution for an RIO/RTO/RIO heterostructure at different temperatures and with four typical H_{loc}/J_{eff} values. $H_{loc}/J_{eff} = 1.4$ is a typical value in the regime of $H_{loc}/J_{eff} < 2$, where the ground state of RIO adopts a 2-in-2-out rule just like the spin ice. The ground state of the entire heterostructure still adopts the 2-

in-2-out rule just like the spin ice. However, at finite temperature such as 1.5 K, we still have a non-zero density of 2DMG in the RTO layer. Here we have a total sheet density of 0.32 q_mu.c.$^{-2}$. $H_{loc}/J_{eff} = 4.4$ is a typical value for $2 < H_{loc}/J_{eff} < 6$ regime, and also is the realistic value for $Ho_2Ir_2O_7$. In this regime, the RIO adopts a 3-in-1-out fragmentation phase. At the ground state, the heterostructure does not host any 2DMG as all the monopoles are absorbed to the very interface layers. At a finite temperature at 1.5 K, the monopoles "sublimate" from the interface layers and go into the RTO layers to form a 2DMG with a total sheet density of 1.04 q_mu.c.$^{-2}$. $H_{loc}/J_{eff} = 8.9$ is a typical value for $6 < H_{loc}/J_{eff} < 12$ regime, where the RIO layer adopts AIAO in the ground state. Still, there are no 2DMG at the ground state, as all the monopoles are absorbed to the interfaces layers. Again, at a finite temperature like 1.5K monopoles come into the RTO layer and form a 2DMG. $H_{loc}/J_{eff} = 14$ is typical value for $H_{loc}/J_{eff} > 12$, where the RIO layer also form AIAO AFM in the ground state. The H_{loc}/J_{eff} value is so large that the monopole can no longer stay at the very interface layer and form a 2DMG even at the ground state with a maximized sheet density of 4 q_mu.c.$^{-2}$.

Supplementary Note 9: AFM domain dependence of 2DMG for an RIO/RTO/RIO heterostructure

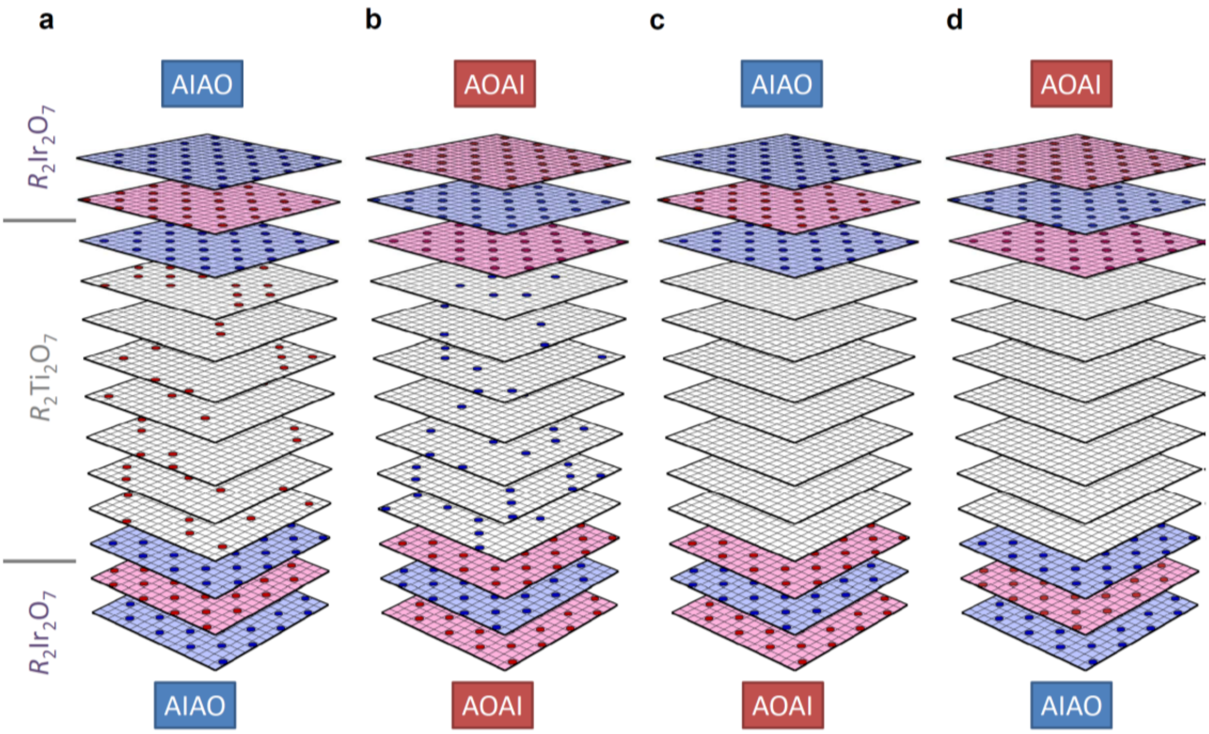

Supplementary Figure 8 | AFM domain dependence of 2DMG for an RIO/RTO/RIO heterostructure Snapshots of monopole distributions at the ground state of an RIO/RTO/RIO heterostructures with AFM domain configuration of **a.** AIAO top and AIAO bottom, **b.** AOAI top and AOAI bottom, **c.** AIAO top and AOAI bottom, **d.** AOAI top and AIAO bottom, with $H_{\mathrm{loc}}/J_{\mathrm{eff}} = 14$.

Just like the 2DEG and the LAO/STO interface, the existence of 2DMG in the RIO/RTO/RIO heterostructures is contingent upon the interface termination. Supplementary Figure 8 shows all four possible terminations for the RIO/RTO/RIO heterostructures, with each interface being all-in layers or all-out layers. Clearly, only when the top interface and the bottom interface have the same terminations, there is 2DMG in the RTO layers. In these cases, the

charge sign of the 2DMG is determined by the interface termination: all-in termination yields positively charged 2DMG, while all-out termination yields negatively charged 2DMG. When the top and bottom interfaces have different terminations, the RTO layers will be a monopole vacuum, with the spin all polarized upwards or downwards.

The AIAO or AOAI domains are determined by the Ir^{4+} moments, which orders at a temperature high than 130 K, where R^{3+} moments are far from being ordered. Also due to the nearest neighbored nature of the exchange interaction between Ir^{4+} moments, top RIO layer and bottom RIO layer are going to order independently. This will result in four possible domain combination as discussed above. Although in reality there has not been an approach to control the domain choice of the RIO materials, one can utilize the microscopy tool to look for the areas with the desired combination of domains.

Supplementary Note 10: Result of 2DMG of an RIO/RTO hetero-interface with NN model

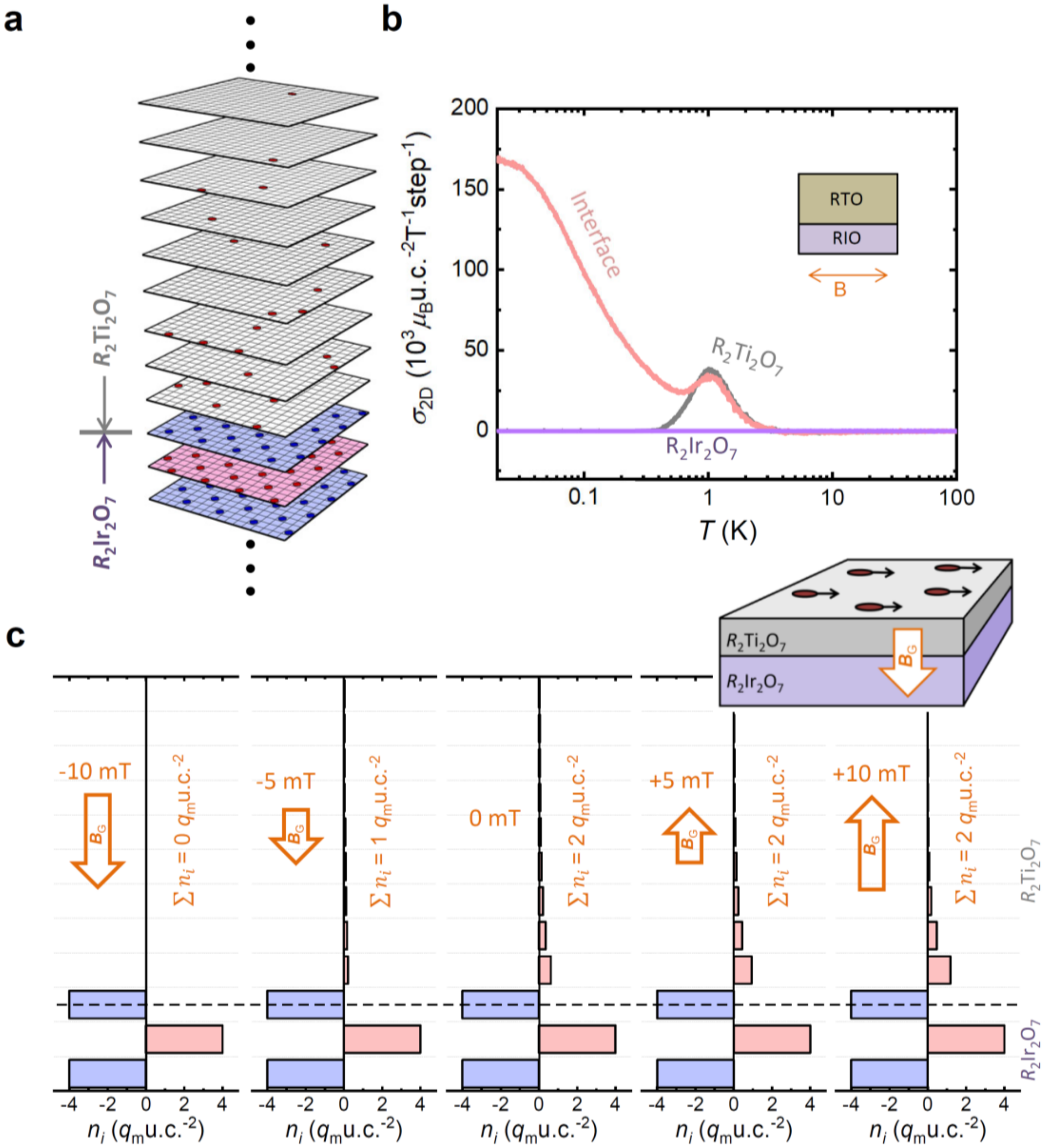

Supplementary Figure 9 | Monte Carlo result of the 2DMG at an RIO/RTO interface a. Snapshots of monopole distributions at the ground state of an RIO/RTO interface with H_{loc}/J_{eff} = 14. **b.** In-plane AC sheet monopole conductivity at the frequency of 1/20 Monte Carlo steps as a function of temperature for the 2DMGs at the RIO/RTO interface as well as

the in-plane AC sheet conductivity of the monopoles for a pure RIO material and a pure RTO material as references at $T = 0.1$ K. **c.** Monopole distribution of the 2DMG of the RIO/RTO interface under different magnetic gate at $T = 0.1$ K.

2DMG also exist at the RIO/RTO interface. Here we modeled a bilayer structure with 3 u.c. thick RIO and 27 u.c. thick RTO. The other side of the RTO is set to be free surface. To study the properties of the interface without mixed by those from the free surface, we just sampled the information for the 3u.c. thick RIO and first 9 u.c. of RTO close to the interface. Just like LAO/STO system, in reality the thickness of RTO can be mm-thick, so that the free surface is well decoupled from the interface.

Supplementary Figure 9**a** shows a snapshot of such an interface at $H_{\mathrm{loc}}/J_{\mathrm{eff}} = 14$, which is in a regime of metallic 2DMG. The monopoles are denser close to the interface and dilute out toward the interior of RTO layers. The majority of 2DMG lives within the first 1-2 u.c. of the RTO layers (one unit cell has four tetrahedral layers). The total sheet density is 2 q_{m}u.c.$^{-2}$, exactly half of that of an RIO/RTO/RIO heterostructures. The transport properties of the 2DMG is shown in Supplementary Figure 9**b**. Clearly it is metallic, in contrast to the insulating behavior of both RIO and RTO pure materials.

Supplementary Figure 9**c** shows the monopole distribution of the 2DMG under different magnetic gate, simulated at $T = 0.1$ K. The purpose of the finite temperature is to prevent the 2DMG being modulated by any infinitesimal gating field. In the negative gate, the 2DMG

can be depleted. In the positive gate, the 2DMG can be accumulated to a maximum sheet density of 2 q_mu.c.$^{-2}$. Further increase gate does not increase 2DMG density, but pushes the 2DMG toward the interface.

Supplementary Note 11: H_{loc}/J_{eff}-T phase diagram of a T-type RIO/RTO/RIO (111) sandwich with NN-only model.

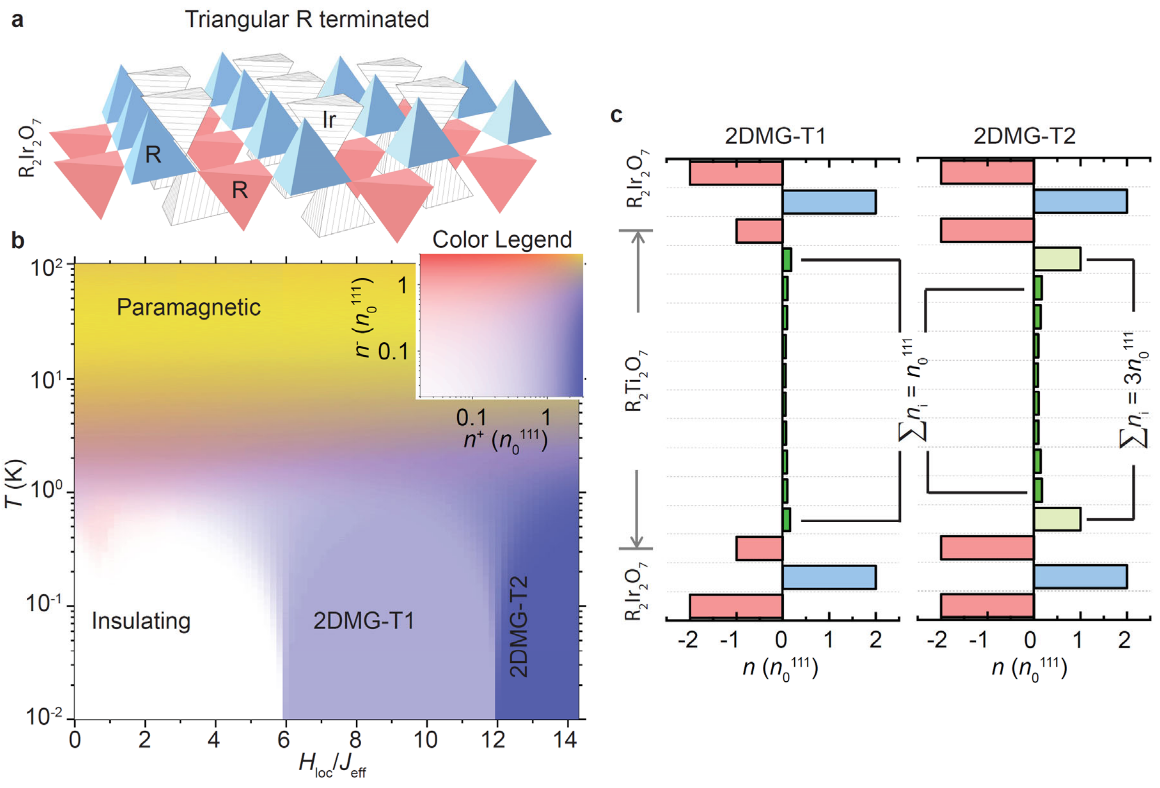

Supplementary Figure 10 | H_{loc}/J_{eff}-T phase diagram of a T-type RIO/RTO/RIO (111) heterostructure with NN-only model. a. Illustration of the lattice structure of a T-type RIO/RTO (111) interface which are terminated with triangular lattice of R^{3+} moments on the iridate side. **b**. Monopole (antimonopole) sheet density $n^{+}(n^{-})$ of the sandwich. Two distinct 2DMG phases are observed, as characterized by different monopole sheet densities in the RTO slab. **c**. Monopole distribution profiles of both 2DMG phases at the ground state.

The T-type sandwich (defined as triangular termination at iridate side) shows a qualitatively similar phase diagram to that of a K-type sandwich (discussed in the main article, defined by Kagomé termination), also characterized by two distinct 2DMG phases: 2DMG-T1 and 2DMG-T2, which exhibit similar monopole distribution to that of the 2DMG-K1 and K2

phase. The H_{loc}/J_{eff} values of the phase boundaries of the T-type sandwich is twice as much as those of a K-type sandwich.

Supplementary Note 12: H_{loc}/J_{eff}-T phase diagram of a RIO/RTO/RIO (001) sandwich with dipolar interaction model

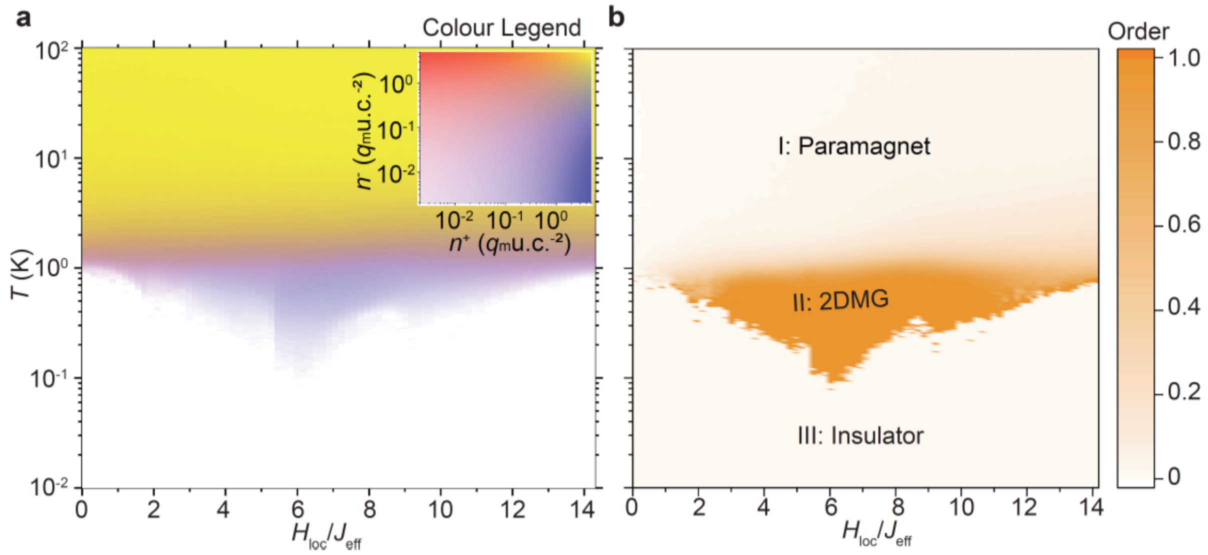

Supplementary Figure 11 H_{loc}/J_{eff}-T phase diagram of a RIO/RTO/RTO(001) heterostructure simulated by Monte Carlo method using the dipolar model. a. the phase diagram of the monopole and anti-monopole sheet density n^+ and n^- in the RTO slab, where the color encodes both densities at the same time. **b**. the phase diagram of the monopole polarity order defined by $p_m = (n^+ - n^-)/(n^+ + n^-)$. The phase diagram is characterized by three regions: the paramagnetic region where RTO is filled with thermal excited monopole-antimonopole pairs; the 2DMG region where the RTO is dominated by one sign of the monopoles; and the insulator region, where RTO is in Coulomb phase.

Supplementary Figure 11**a** shows the H_{loc}/J_{eff}-T phase diagram of the monopole and anti-monopole sheet density in a RIO/RTO/RIO (001) sandwich with dipolar interaction model. Similar to the case without long ranged interactions, the system is paramagnetic at high temperature, and shows a net charge near 1K, at all H_{loc}/J_{eff} values. However, further cooling

down the system toward the ground state leads to the insulating phase, for all H_{loc}/J_{eff} values. Supplementary Figure 11 **b** shows the monopole polarity order p_m, defined as $p_m = (n^+ - n^-)/(n^+ + n^-)$. Clearly, for nearly all H_{loc}/J_{eff} values, there always exists a finite temperature window where p_m is almost 1. This means that we can always find a temperature window where the 2DMG is nearly singly signed.

Supplementary Note 13: The proposal an AFM/spin ice interface in an artificial system.

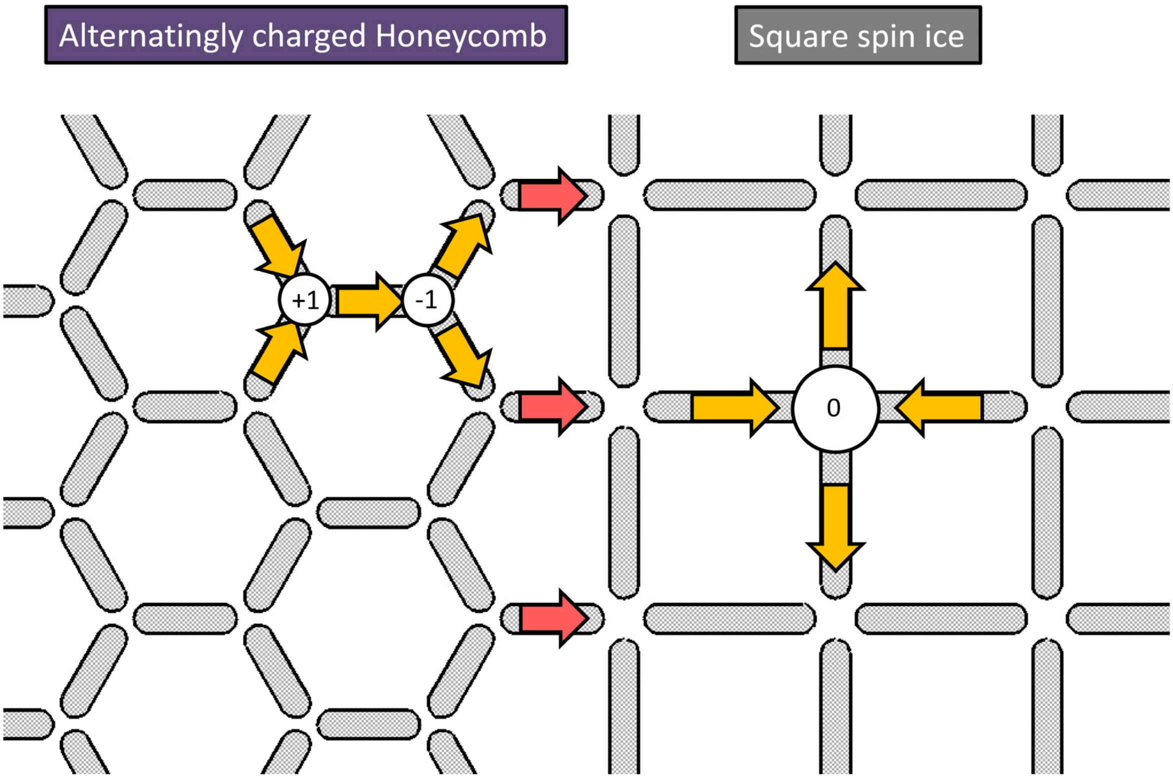

Supplementary Figure 12 | Illustration of an AFM/spin ice heterostructures in the artificial spin system An interface between a honeycomb lattice and a square lattice, where the honeycomb lattice plays the role of the AFM, and the square lattice plays the role of the spin ice.

Here we show that the idea of realizing the monopole gas in AFM/spin heterostructures can also be applied to the artificial sin ice system. Supplementary Figure 12 shows the structure of an interface between a honeycomb lattice and a square lattice. The lattice is made of ferromagnetic rods via nanofabrication. Due to the dipolar interaction between the rods, each junction favors a 2-in-2-out rule and carries a zero magnetic charge for the square lattice, resembling the spin ice material, while the junction in the honeycomb favors a 2-in-1-out or 2-out-1-in rule. Due to the odd number of rods per junction, each junction must be

magnetically charged. Also due to the dipolar interaction, a positive charge favors a negative charge at the neighboring position, as shown in Supplementary Figure 12. Since there are two types of inequivalent sites in honeycomb lattice, each type of sites will adopt a type of charge, forming two possible domains, in which the layers of the sites are alternating charged by positive 1 or negative 1. In this way the honeycomb resembles the RIO material. Given that there is also a polar discontinuity at the interface between the honeycomb lattice and the square lattice, one should expect a one dimensional charged monopole gas exist in the squared layers, close to the interface. This system will be a good platform to visualize the charged monopole gas in the real space.

Supplementary References

[1] Lefrançois, E., Cathelin, V., Lhotel, E., Robert, J., Lejay, P., Colin, C.V., Canals, B., Damay, F., Oliver, J., Fåk, B., Chapon, L.C., Ballou, R., and Simonet, V., "Fragmentation in spin ice from magnetic charge injection", *Nat. Commun.* **8**, 20 (2017).

[2] Matsuhira, K., Wakeshima, M., Hinatsu, Y., and Takagi, S. "Metal-insulator transitions in pyrochlore oxides $Ln_2Ir_2O_7$", *J. Phys. Soc. Jpn.* **80**, 094701 (2011).

[3] Ma, E.Y., Cui, Y.-T., Ueda, K., Tang, S., Chen, K., Tamura, N., Wu, P.M., Fujioka, J., Tokura, Y., and Shen, Z.X. "Mobile metallic domain walls in an all-in-all-out magnetic insulator", *Science* **350**, 538 (2015).

[4] Bovo, L., Moya, X., Prabhakaran, D., Soh, Y.-A., Boothroyd, A.T., Mathur, N.D., Aeppli, G., and Bramwell, S.T. "Restoration of the third law in spin ice thin films", *Nat. Commun.* **5**, 3439 (2014).

[5] Jaubert, L.D.C., Lin, T., Opel, T.S., Holdsworth, P.C.W., and Gingras, M.J.P. "Spin ice film: surface ordering, emergent square ice, and strain effects" *Phys. Rev. Lett.* **118**, 207206 (2017).

[6] Melko, R.G., den Herthog, B.C., and Gingras, M.J.P. "Long-range order at low temperatures in dipolar spin ice" *Phys. Rev. Lett.* **87**, 067203 (2001).

[7] Melko, R.G. and Gingras, M.J.P. "Monte Carlo studies of the dipolar spin ice model" *J. Phys. Condens. Matter.* **16**, R1277 (2004).
