# Supplementary material for: Two-dimensional magnetic monopole gas in an oxide heterostructure
Source: Nat Commun. 2020 Mar 12;11:1341. doi: 10.1038/s41467-020-15213-z (PMC7067881; doi:10.1038/s41467-020-15213-z)
Supplement: Supplementary file 2 — Peer Review File [file 41467_2020_15213_MOESM2_ESM.pdf]

Editorial Note: This manuscript has been previously reviewed at another journal that is not operating a transparent peer review scheme. This document only contains reviewer comments and rebuttal letters for versions considered at Nature Communications.

REVIEWERS' COMMENTS:

Reviewer #1 (Remarks to the Author):

The authors have addressed all my concerns in detail. I think by focussing purely on the semiconductor analogy the paper is much stronger -- it is very interesting work that should inspire experiments. I recommend it for Nature Communications.

One small comment:

Abstract:
The new sentence" However, unlike semiconductors and two-dimensional electron gases where the charge degree of freedom can be actively controlled by chemical doping, interface modulation, and electrostatic gating, there is as of yet no analogue for emergent magnetic monopoles. "

The sentence is a bit ambiguous as it stands. To be precise, I would suggest inserting "of these effects" after "as yet no analogue".

Reviewer #2 (Remarks to the Author):

Report on the article entitled « Two-dimensional magnetic monopole gas in an oxide heterostructure » by K. Miao et al. Submitted for publication at Nature Communications

The authors have taken into account my comments and they have much improved the clarity of their manuscript. I would only recommend to better explain the figure 1c in the corresponding caption, in particular the truncated Ir4+ hexagons in green and the reduced local field materialized by green arrows. It would also be nice to indicate the values of the colour scale of figure 3Sf.

With these modifications, I recommend publication of this article in Nature Communications. This work indeed clearly demonstrates a very interesting and original idea in order to obtain a two-dimensional magnetic monopole gas in pyrochlore heterostructures. This should undoubtedly foster further experimental works.

Response to Reviewer #1

1. "*The new sentence 'However, unlike semiconductors and two-dimensional electron gases where the charge degree of freedom can be actively controlled by chemical doping, interface modulation, and electrostatic gating, there is as of yet no analogue for emergent magnetic monopoles.' The sentence is a bit ambiguous as it stands. To be precise, I would suggest inserting 'of these effects' after 'as yet no analogue'.*"

We agree with the reviewer that the current sentence is ambiguous. We have added the phrase "*of these effects*" into this sentence, as suggested by the reviewer.

Response to Reviewer #2

1. "*The authors have taken into account my comments and they have much improved the clarity of their manuscript. I would only recommend to better explain the figure 1c in the corresponding caption, in particular the truncated Ir4+ hexagons in green and the reduced local field materialized by green arrows.*"

We apologize for the unclear explanation of Figure 1**c**. We have added "*The complete/truncated hexagons ... are materialized by green arrows.*" into the figure caption as suggested by the reviewer.

2. "*It would also be nice to indicate the values of the colour scale of figure 3Sf.*"

We thank the reviewer for pointing this out. The Supplementary Figure 3 does not involve color plot which requires a color scale. But we indeed missed color scale in the Supplementary Figure 6. We have now added color scales with indicated values to all the panels in Supplementary Figure 6 to address the reviewer's comment. And we have checked the main article and Supplementary Information to ensure that all the color encoded data figures have a color scale with indicated values.

.
